# Comparative Seroepidemiological Study of Brucellosis in Sheep under Smallholder Farming and Governmental Breeding Ranches of Central and North East Ethiopia

**DOI:** 10.1155/2018/7239156

**Published:** 2018-07-04

**Authors:** Shimeles Abegaz Addis, Andualem Yimer Desalegn

**Affiliations:** School of Veterinary Medicine, Wollo University, P.O. Box 1145, Dessei, Ethiopia

## Abstract

This cross-sectional study was carried out to investigate the status of brucellosis in sheep management under extensive smallholder farming and intensively in governmental breeding ranches in six districts selected from three administrative zones. Using multistage sampling, serum samples of 2409 sheep from 274 flocks were collected and tested using the Rose Bengal Plate Agglutination Test (RBPT) and positive sera were confirmed using a Complement Fixation Test (CFT). Of all animals tested, 4.98% were RBPT positive, and after confirmation with CFT, the overall animal-level prevalence was found to be 4.89% (CI: 3.24-6.9%). Of the flocks sampled, 61 (22.3%, CI: 18.03-29.17%) had at least one animal positive to both tests. Significantly higher (*P* < 0.001) individual animal seroprevalence of 5.87% (CI: 3.83-7.31%) was found in sheep under smallholder production than in breeding ranches (1.75%, 95% CI: 1.57-3.05%). However, flock level seroprevalence in breeding ranches was found to be 100% (8/8), while in the smallholder production it was 19.92% (CI: 16.4-25.81%). Significantly highest animal-level seroprevalence of 9.55% (CI: 7.91-12.4%) was observed in north Wollo zone's smallholder farms. From the three studied breeding ranches, highest seroprevalence of 3.57% (CI: 2.84%-5.18%) was found in Sheno Agricultural Research Centre. Significantly higher seroprevalence (*P* < 0.01) was found in aborted sheep and with history of retained fetal membrane in both production systems. All the sheep flocks in the studied breeding ranches were found to be seropositive; hence, this study suggests strict control measures of ovine brucellosis in the breeding reaches, since they could be a source of infection for the smallholder farms.

## 1. Introduction

Brucellosis is a zoonotic disease that has a significant economic, social, and public health impact in many parts of the world. Brucellosis is caused by the bacteria of the genus* Brucella* and Gram-negative intracellular coccobacilli, which occur in a wide variety of animals including cattle, sheep, goats, pigs, and other livestock as well as humans [1]. Nine* Brucella* species are currently recognized; seven of them that affect terrestrial animals are* Brucella abortus*,* B. melitensis, B. suis, B. ovis, B. canis, B. neotomae*, and* B. microti *and the two other species that affect marine mammals are* B. ceti* and* B. pinnipedialis *[2].

In small ruminants, significant reproductive losses are usually caused by* Brucella melitensis *and* Brucella ovis*. Although the prevalence of this disease varies widely from country to country, small ruminant brucellosis is mostly caused by* B. melitensis* and remains one of the most important zoonotic diseases [3]*. Brucella ovis* is a nonzoonotic species, which is an important cause of orchitis and epididymitis in sheep, but it is not recognized as a cause of natural infection in goats [4].

Brucellosis spreads between animals in a herd and the disease is a systemic infection that can involve many organs and tissues. Persistent infection is a common feature of the disease with frequent shedding of the bacterium in reproductive and mammary secretions. The brucellosis can have a considerable impact on human and animal health, as well as socioeconomic impacts, especially in which income relies largely on livestock breeding. Furthermore, in animals, brucellosis causes severe economic losses as result of stormy abortions or reproductive failure, sterility, and reduced milk production rates. Also brucellosis of animals reduces the foreign exchange earnings (FEE) by denying exportation of sheep to international markets [5, 6].

Isolation and identification of the causative agent from abortion material and udder secretions or from tissues removed at postmortem constitute the most preferred method for presumptive diagnosis of brucellosis. However, bacteriological diagnosis is hazardous and time-consuming and is not a practical and reliable means for diagnosis in large-scale programs [7]. Consequently, serological tests based upon the detection of* Brucella* antibodies against the organism are the most useful epidemiological tool for laboratory diagnosis of* Brucella* infection [8]. The RBT and the CFT are the most widely used tests for the serodiagnosis of brucellosis [9]. Usually, the RBT is used as a screening test and CFT as a confirmatory test based on an antigen reaction of the entire cells of* Brucella* and the antibodies produced as response to infection [9].

Although eradicated in many developed countries after years of effort, brucellosis is endemic in Africa [10] and also remains a major neglected zoonosis of low-income nations [5]. Factors related to the host*‚* the agent*‚* the environment, and management practices determine the extent of exposure*‚* spread, and maintenance of brucellosis in a geographical area [11]. The spread of an infection from country to country or within the same country generally follows the transfer of infected animals. Brucellosis is also transmitted from farm to farm through wild animals and dogs responsible for carrying around aborted fetuses [12]. Mixing herds at pasture and keeping the animals in shelters during the night, particularly if in such areas parturition takes place, represent major factors for transmission of the infection, especially in sub-Saharan Africa, where these circumstances are commonly found [13, 14].

Ethiopia with over 85% of its population engaged in agricultural activity livestock plays a significant role, directly or indirectly, in achieving food self-sufficiency. Of the total household cash income from crop and livestock, livestock account for 37 to 87% in different parts of the country [15]. In the central highlands of Ethiopia, where mixed crop-livestock production system is practiced, small ruminants account for 40% of cash income and 19% of the household meat consumption [16]. There is also a growing export market for sheep and goats meat in the Middle Eastern Gulf states and some African countries [17]; however, occurrence of infectious and economically important animal diseases in Ethiopia excludes the country from profitable international markets, thereby greatly reducing the country's foreign exchange earnings [18].

The country has diverse agroecological zones, which have contributed to the evolution of different agricultural production systems. The huge and diverse livestock species in Ethiopia are kept under different agroecological zones, production systems, and migration and animal health care system. The predominant extensive husbandry practices of the country provide ample opportunities for mixing multiple livestock species per holding; stock density and social organizations to handle livestock may account for the widespread risk factors for maintenance and transmission of brucellosis [19].

More importantly, a close human-animal contact and tradition of raw animal product consumption make zoonosis among the major public health hazards. This requires thorough epidemiological investigations including due consideration to identify the major risk factors that predominantly influence the disease occurrence and thus contribute to designing appropriate and feasible national controlling strategies. The existence of brucellosis has been confirmed and reported by various workers in different animal species in Ethiopia. Most of the previous studies on small ruminant brucellosis have been carried out in eastern Ethiopia, where pastoral production is practiced, and do not provide an adequate epidemiological picture of the disease in different agroecological zones and livestock production systems of the country. In particular, there is no information on sheep brucellosis in governmental breeding ranches of Amhara regional state. Therefore, the objective of the present study was to determine the seroprevalence of sheep brucellosis in two production systems in smallholder extensive and intensive breeding ranches and to point out the associative factor for the occurrence of the disease.

## 2. Materials and Methods

*Ethical Approval*. All essential procedures of sample collections were performed strictly as specified by Institutional Ethics Committee with minimal stress to animals.

### 2.1. Study Area

**Figure 1:**
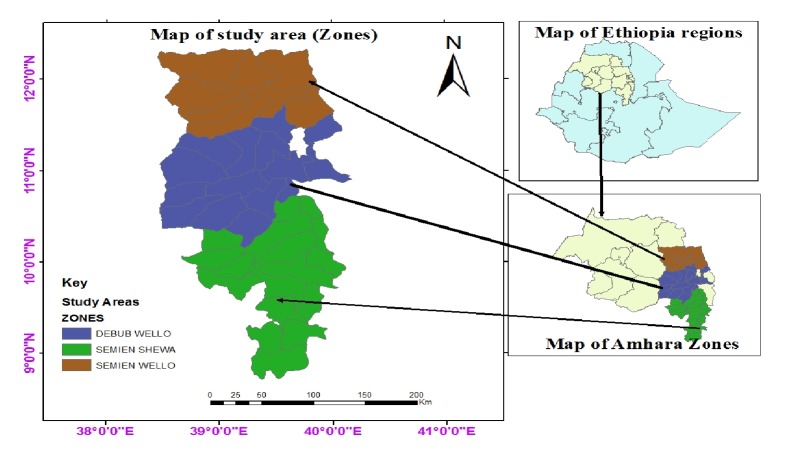
Map of Ethiopia, Amhara regional state, and the study zones.

This cross-sectional study was conducted in selected districts of south Wollo, north Wollo, and north Shewa administrative zones of the Amhara regional state (Figure 1) from December 2014 to June 2015. The Amhara region is located in the northwestern part of Ethiopia between 9°20′ and 14°20′ north latitude and 36°20′ and 40°20′ east longitude. The region is border with Tigray, Afar, Oromia, and Benishangul-Gumuz regions of the country (Figure 1) and with Sudan in the west of the region. The altitude of the areas ranges from 1800 to 2900 meters above sea level and experiences a bimodal rainfall pattern with a short rainy season from February to March and the long rainy season, which starts at the end of June and ends at the end of September [20]. The annual rainfall of the areas ranges from 850 to 1100 mm and the mean annual minimum and maximum temperatures are 7.8°C and 21°C, respectively [20, 21]. The area is marked by two distinct seasons, the dry season extending from December to May and the wet season that extends from June to September. Traditionally, agriculture including livestock farming is the livelihood of major section of the population in Amhara region, and this is characterized mostly by crop-livestock mixed farming. Sheep are the predominant species, kept under smallholder extensive farming in the areas. The areas are dominated by small-scale farming, most commonly with <20 sheep and 1–3 cows per household. Sheep are most commonly used for meat production. An average-sized peasant association (PA) in the study area has approximately 100 households, and these urban villages in the PAS often have access to vast pastures, where communal grazing is common throughout the year.

There are three governmental sheep breeding ranches, Sheno Agricultural Research Centre, Amedguya, and Debre Berhan breeding ranches, in eastern Amhara regional state. Those breeding ranches are found in north Shewa administrative zone of the state established with major objective of local/indigenous sheep breeds improvement by crossing with exotic breeds. Awassi sheep breed is the main breed raised in the breeding ranches, which is considered as a good breed for meat production. Sheno Agricultural Research Centre is the first established breeding ranch for improvement of the indigenous Menz sheep with the Awassi breed. The Awassi breed sheep were imported from Israel to Ethiopia and were crossed with the indigenous Menz breed [22]. These crosses of Awassi-Menz have been well accepted by farmers of Ethiopian highlands and, subsequently, producing Awassi-Menz cross breeds has been boosted by establishing another ranch, Amedguya breeding ranch, at distance of 300 km northeast of Addis Ababa. Cross breed rams from those breeding ranches are currently distributed to smallholder farmers on a cost-recovery basis all over the region.

### 2.2. Study Population

The study animals were indigenous breeds of sheep kept under smallholder extensive management system and sheep managed in government breeding intensive ranches. The smallholder farming is characterized by crop-livestock mixed farming system in which animals graze communally and return in the evening but without supplementary feeds. Breeding ranches are intensively managed and usually fenced and the sheep are well supplemented with feeds in addition to natural pastures. Sheep that are older than 6 months of age with no history of vaccination against brucellosis were included in the study. Data collected for each individual animal were age, sex, breed, number of sheep owned, history of abortion/stillbirth, and name of the district and village.

### 2.3. Study Design and Sampling Strategy

A cross-sectional epidemiological study was conducted from December 2014 to June 2015 to determine seroprevalence of brucellosis in sheep from three selected geographical zones of Amhara regional state and to identify factors associated with seropositivity. The study population was stratified into two strata based on the management system, sheep flocks in government breeding ranches and smallholder extensive farms. The stratification was required because the study population is kept under different management types, which could influence the prevalence of brucellosis. A multistage stratified random sampling method was used in the current survey as described by Martin et al. [23] and Thrusfield [24].

### 2.4. Selection of Study Districts and Peasant Associations (PAS)

Amhara regional state is divided into 11 administrative zones and 140 districts. Three administrative zones (south Wollo, north Wollo, and north Shewa zones) were selected purposively based on their location, proximity to a reliable laboratory, farming systems (breeding ranch and smallholder farms), sheep populations, cooperation from farmers, and contribution of sheep for export market. Six districts were randomly selected from Amhara regional state, two districts per administrative zone, using as sampling frame a list of all districts in each zone. From each of the six selected districts, the peasant associations (PAS) included/selected had to be located with radius of less than 25 km from the center of each district to be accessible by car. Another inclusion criterion was information on the PAS keeping sheep (sheep population). The PAS that met the inclusion criterion were listed for each district. Three to five peasant associations (PAs) were randomly selected from each district using as sampling frame a list of all PAS in each district, which were made available by district livestock officers.

### 2.5. Sampling Procedure for Smallholder Farms/Flocks

Multistage sampling technique was used according to Dohoo et al. [25] in the survey of sheep brucellosis kept under smallholder extensive farms. From each of the six selected districts, 3-5 peasant associations (PAs) were randomly selected. Proportion sampling was adopted to obtain the number of flocks from each of the selected PAS per study district. Since no sampling frames were available for selection of flocks within PAS, the selection of flocks within each PAS was performed on site and based on whether the householder was present and willing to participate in the study. Within each PAS, 8-15 flocks were randomly selected as the primary sampling units and individual sheep as secondary units. Within each flock, sheep were selected by simple random sampling. A maximum of 14 individual sheep that met the inclusion criteria were sampled per flock.

### 2.6. Sampling Procedure for Breeding Ranches

There are three government sheep breeding centers (ranches) in north Shewa zone of Amhara regional state. The adult sheep populations in all ranches were recorded and then around 34% of the adult population from each ranch was sampled using systematic random sampling. The sampling frame was comprised of individual animals from farm records and individual sheep was sampled using systematic random sampling at interval of two sheep starting from the first animal from individual sheep records of each breeding ranch.

### 2.7. Sample Size

A total of 2409 sheep sera were collected to examine the presence of antibodies against* Brucella* organism. A two-stage cluster sampling technique has been used to calculate the minimum sample size. The sample was collected from sheep kept under different management system: in breeding intensive ranches (*n* = 571) and smallholder farms (*n* = 1838). The sample size for smallholder farms was determined using standard procedures as described by [24] for an infinite population. In order to determine the desired sample size, there were no previous reports of prevalence in the selected zones. Hence, the average expected prevalence rate was assumed to be 50% for the area within 95% confidence intervals (CI) at 5% desired accuracy; thus the desired minimum sample size is *n* = 384 per each zone with total samples of 1152 sheep to be sampled for serological studies but due to the interest and cooperation of smallholder farmers inconsideration with available logistics and resources, we were able to sample a total of 1838 sheep from six districts that have higher sheep population in the region. The number of animals sampled from each area was proportionally distributed based on the total sheep population in the study zones, districts, and PAs. Hence, from north Shewa zone (*n* = 446), south Wollo zone (*n* = 901), and north Wollo zone (*n* = 459), sheep were sampled.

### 2.8. Sample Collection

Approximately 8-10 ml of blood samples was collected from jugular vein of each sheep of selected flocks aseptically using sterile plain vacutainer tubes and needles. Individual tubes were identified using numbers and alphabets to indicate their origin. The tubes were left tilted over night at room temperature to allow clotting. The sera were separated from clotted blood (unretracted blood being centrifuged) by siphoning into sterile Eppendorf tubes. The samples were shipped to the Kombolcha Animal Health Diagnostic Laboratory in icebox and stored at –20°C until serological testing was performed.

### 2.9. Serological Tests

*Rose Bengal Plate Test (RBPT)*. The Rose Bengal Plate Test was used as a screening test of serum samples for the presence of* Brucella *antigens. All blood samples collected were first screened using Rose Bengal Plate Test (RBPT) at Kombolcha Regional Veterinary Laboratory, following the procedure described by Neilsen and Dunkan [26]. The sera and RBPT antigen, which constitute a suspension of* Brucella abortus* with cross-reaction against* B. melitensis* antibodies in serum samples (Institut Pourquier 325, rue de la Galèra 34097 Montpellier, Cedex 5, France), were taken out of the refrigerator and left at room temperature for at least 30 minutes before the test was performed. Briefly, 30 *μ*l of the sera samples was dispensed onto the plate and 30 *μ*l of RBPT antigen was dropped alongside the sera. Using applicator stick, the antigen and the sera were mixed and examined for agglutination. The antigen presentation was 10 ml of RBPT antigen per bottle. For interpretation of results, both positive and negative controls were employed; agglutination reactions were read in a good light source or using a magnifying glass when microagglutinations were suspected. Results of RBPT were interpreted as 0, +, ++, and +++ as has been described by [25], where 0 indicates no agglutination, + indicates barely visible agglutination (using magnifying glass), ++ indicates fine agglutination (some clearing), and +++ indicates clumping, definite clearing. Those samples identified with no agglutination (0) were regarded as negative, while those with +, ++, and +++ were regarded as positive.

*Complement Fixation Test (CFT)*. Positive sera with RBPT were further tested with CFT for confirmation using standard* Brucella abortus *antigen (New Haw, Addlestone, Surrey KT15 3NB, UK). CFT was conducted at the National Veterinary Institute, at Debre Zeyit. The CFT test's proper and reagent preparation procedures were following the procedures outlined by OIE [27]. Sera with strong reaction or more than 75% fixation of complement (3+) at a dilution of 1:5 or at least with 50% fixation of complement (2+) at a dilution of 1:10 and above were classified as positive [27].

### 2.10. Data Analysis

All the collected data were stored in a spreadsheet in the Microsoft Excel program. Epidemiological and statistical analyses were performed as is required by using SPSS version 20. Chi-square test was used to determine presence of association of different risk factors with that of seropositivity to* Brucella* antibody and 95% confidence interval (CI) at 5% cut-off value was set for significance. For the proportions, the 95% confidence interval (95% CI) was estimated using the exact binomial test [28]. The data were compiled by summing up the laboratory findings and field observation into districts and zones units accordingly. An animal was said to be positive if it tested positive to both RBPT and CFT. A flock having at least one seropositive sheep was considered as positive.

## 3. Results

At the individual animal level, a total of 2409 sera samples were tested with Rose Bengal Plate Test (RBPT), of which 120 (4.98%) sheep were positive for brucellosis. The RBPT positive serum samples were confirmed by complement fixation test (CFT) with 118 (4.89%, 95% CI: 3.24-6.9%) of the samples being seropositive for brucellosis upon further testing by CFT. Based on test agreement analysis by KAPA test, all RBPT negative sera were CFT negative, but 98.33% (118/120) of those that were found to be positive by RBT were found to be positive by CFT (Table 1). The agreement between RBPT and CFT to detect* Brucella* infection was excellent (*k* = 0.958)

The prevalence of flocks with at least one seropositive sheep for brucellosis was estimated to be 22.3% (95% CI: 18.03-29.17). Of the total 61 seropositive sheep flocks, 53 flocks were in smallholder extensive production, while all the 8 sheep flocks examined in breeding ranches were found 100% (8/8) sero-positive for brucellosis (Tables 2 and 3).

According to the present study, significantly higher (*P*< 0.001) individual animal sero-prevalence of 5.87% (95% CI, 3.83-7.31) was found in sheep under smallholder extensive production system than in breeding ranches with prevalence of 1.75% (95% CI: 1.57-3.05) (Table 2). Flock level seroprevalence of ovine brucellosis was 19.92% (95% CI: 16.4-25.81 in the extensively managed smallholder sheep flocks), while all the eight sampled flocks from breeding ranches were 100% seropositive. For the flock level seroprevalence of ovine brucellosis, there was a statistically significant difference (*P* < 0.01) among the two production systems (Table 3).

The seroprevalence of ovine brucellosis among the three administrative zones at individual level and flock level was significantly different (*P* < 0.001). The highest individual animal seroprevalence was found in north Wollo, 9.55% (95% CI: 7.91-12.4 %), followed by 6.1% (95% CI: 4.83-7.41%) in south Wollo and 2.3% (95% CI: 1.02-3.51%) in north Shewa (Table 4).

The lowest flock level seroprevalence of brucellosis was observed in north Shewa zone, 7.91% (95% CI: 5.21-10.0), whereas almost similar seroprevalence of 33.3% and 32.69 % was found in south Wollo and north Wollo zones, respectively (Table 4). There was no statistically significant difference in flock level seroprevalence between south Wollo and north Wollo (*X*^2^ = 6.02, df = 1, and* P* > 0.05).

Significantly higher (*P* < 0.01) seroprevalence was observed in animals from midland agroecology, 9.07% (95% CI: 7.54-11.7%), compared with the highland sheep (4.5%, 95% CI: 2.71-5.03). Similarly, prevalence of 29% and 16.9% (*P* < 0.05) was recorded in highland and in midland sheep flocks, respectively (Table 4).

The animal level seroprevalence of brucellosis was significantly different among the three studied sheep breeding ranches. Higher prevalence of 3.57% (95% CI: 2.84–5.18) was observed in Sheno Agricultural Research Centre breeding ranch and it was 2.33% (95% CI: 2.01–2.57) and 1.71% (95% CI: 1.34–2.20) in Debre Berhan and Amedguya breeding ranches, respectively (Table 5).

**Figure 2:**
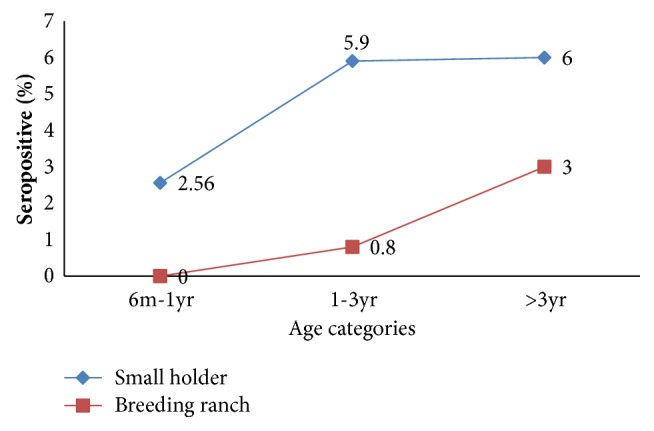
Seroprevalence of brucellosis in terms of age category in breeding ranches and smallholder production.** m: month; yr: year**.

Age of the sheep was categorized into three groups: 6 months to 1 year (*n* = 78), 1-3 years (*n* = 617), and above 3 years (*n* = 1143). Among the age groups, the highest seroprevalence was 6% for those above three years, 5.9% for those between 1 and 3 years, and 2.56% for those between 6 months and 1 year in the extensive production system. Seroprevalence significantly increased in age groups of 6 months to 1 year and 1-3 years (*X*^2^ = 34,* P* < 0.05); however, almost similar seroprevalence was observed between age categories of 1-3 years (5.9%) and above 3 years (6%) (Figure 2). In the intensive production system significantly different (*P* < 0.05) seroprevalence of 0% in 6 months to 1 year group, 0.8% in 1-3 years group, and 3.1% in above 3 years group was (Figure 2).

The seroprevalence was not significantly different (*P* > 0.05) between female and male sheep in breeding ranches. But the prevalence was relatively higher in females (1.93%, 95% CI: 1.01-4.63) as compared to males (0.95%, 95% CI: 0.42-3.83%), although the number of male sheep examined was lower due to the low number of male breeding animals kept in the study breeding ranches. However, in smallholder production system, significantly higher (*P* < 0.001) seroprevalence was observed in female (8.21%, 95% CI: 5.2-11.8) than in male sheep (3.01%, 95% CI: 2.41-4.34) (Table 6).

The seroprevalence of ovine brucellosis was significantly different among the three flock size categories (*P* < 0.001). Seroprevalence in larger flock size with more than 20 sheep per flock (37%) was higher than that in medium-size flocks with 11-20 sheep and in smaller flock with less than 10 sheep per flock (10%) (Table 6).

The seroprevalence of brucellosis in ewes was significantly associated with history of abortion (40%, 95% CI: 32.1-52.4,* P* < 0.01) and retained fetal membrane (50%, 95% CI: 36.12-64.7,* P* < 0.01) compared with none aborted (15.6%, 95% CI: 12.4-18.71) and no fetal membrane (17%, 95% CI: 13.4-25.3) in the extensive smallholder ewes (Table 7). Similarly, seropositivity of ewes for brucellosis was significantly associated with history of abortion (6.25%,* P* < 0.001) and retained fetal membrane (8.3%,* P* < 0.01) in breeding ranches (Table 7).

## 4. Discussion 

In the present study, flock level and individual animal prevalence of ovine brucellosis were estimated in three selected administrative zones of Amhara National Regional State (ANRS) using a probability sampling frame work and RBPT and CFT as a diagnostic tests. Off the total 2409 sera samples collected and screened by RBPT (Rose Bengal Plate Test), 4.98% (*n* = 120) were seropositive for brucellosis. Then, those 120 positive serum samples by RBPT were further tested by CFT for confirmation and 118 (4.89%) samples were positive for brucellosis. When CFT results were compared with those in RBPT, 118 samples were shown as positive in both tests. Statistical analysis of the results demonstrated an excellent agreement between RBPT and CFT test results because the test agreed 95.8% of the time to detect ovine brucellosis (Table 1). The CFT confirmatory test reduced the number of positive animals from 120 to 118 with false positive result of 2/120 (1.67%). This may be due to CFT elimination of some reactions due to cross-reacting bacteria. The RBPT is susceptible to cross-reaction with other Gram-negative bacteria such as* Yersinia enterocolitica*, O:9,* E. coli*, O:157, and also some* Salmonella* spp., which could lead to false positive results [9]

The current higher test agreement result (95.8%) between RBPT and CFT indicated that there has been very active* Brucella* infection in the study areas and agrees with the previous work of Mohammed [29] who reported seroprevalence of brucellosis of 1.64% and 1.51% using RBPT and CFT, respectively, with 95% test agreement in test positivity. Based on this study,* Brucella* antibody was found to be widely distributed in indigenous sheep in the study areas with seroprevalence of 4.89%; out of these seropositive sheep, 75% gave very strong reaction (4^+^ and 3^+^) to CFT, while 25% gave 2^+^ reaction.

According to the present study, the overall individual animal level and flock level seroprevalence of ovine brucellosis was 4.89% (95% CI: 3.24-6.9) and 22.26% (95% CI: 18.03-29.17), respectively. The overall individual level seroprevalence of ovine brucellosis in this result was comparable with previous reports by different researchers, 4.4% in central Ethiopia by Deddefo et al. [30], 5% in India by Vipan Kumar et al. [31], 5.2% in Nigeria by Bertu et al. [32], and 4.2% in Iran by Akbarmehr and Ghiyamirad [33]. Higher individual level seroprevalence rate of ovine brucellosis in sheep compared to this finding was reported, 7.1% by Wesinew et al.[34], 7% by Negash et al. [35], and 14.6% by Teshale et al. [36] in Afar region, pastoral area of Ethiopia. Similarly, higher prevalence of ovine brucellosis was reported in different neighboring African countries, 6.01% in Kenya, 7.2% in Somalia, and 14% in Khartoum, Sudan [37]. However, the animal level seroprevalence obtained in this study was much higher than the reports, 1.5% by Tekelye and Kasali [38] in central Ethiopia and 3.2% by Ashenafi et al. [39] in Afar region of the country. Similarly, very low level of ovine brucellosis seroprevalence in sheep was recorded in Nigeria (0.0%) by Cadmus et al. [40] and in Bangladesh (1.3%) by Rahman et al. [41]. In addition, very low seroprevalence of 0.1, 0.4, and 2.1% was reported by Omer et al. [42] in sheep in Sudan.

The variation in seroprevalence of ovine brucellosis discussed above in different areas may be related to the fact that prevalence of brucellosis may vary depending upon the breed involved, herd size, management, and seasonality of the disease, which could affect the rate of transmission of* Brucella* infection. Supporting the above idea, Acha and Szyfres [43] reported that the rate of* Brucella* infection varies greatly from one country to another and also between regions even within a country.

In contrast to our finding, higher seroprevalence rates of ovine brucellosis were reported in Afar region of Ethiopia [34–36] where pastoral production system is commonly practiced compared to current result recorded in the crop livestock mixed smallholder farming system of our study areas. The higher prevalence reports of brucellosis in a pastoral management system may partly be attributed to long distance movement of animals in search of pasture and water, particularly during the dry season. The movement of animals for grazing and watering as aggregating different species of animals around watering point will increase the contact between infected and healthy animals, thereby facilitating the transmission/spread of brucellosis. In mixed smallholder farming system, fewer animals are raised in separate herds.

Of the three administrative zones, significantly higher animal level seroprevalence of 9.55% (95% CI: 7.91-12.46%) of brucellosis was identified in north Wollo zone than in south Wollo (6.1%) and north Shewa (2.3%). At the same time, among the three zones, there was comparable flock level seroprevalence in south Wollo (33.33%) and north Wollo (32.69%); however, significantly lower flock level prevalence (7.9%) was found in north Shewa zone. The higher prevalence in north Wollo and south Wollo zones of Amhara region in the present study might be due to the fact that the two zones are borders to Afar pastoral region and constant transborder movement of animals has been reported especially at dry season. This common practice of pastoralists moving their animals from pastoral areas of Afar region to the nearby zones of Amhara region (north Wollo and south Wollo zones) for searching greasing and water for their animals may result in transmission of brucellosis.

In the present study, significantly higher (*P* < 0.001) animal level seroprevalence of brucellosis, 5.87% (95% CI: 3.83-7.31%), was found in sheep under smallholder extensive production system than in intensive breeding ranches with prevalence of 1.75% (95% CI: 1.57-3.05). In agreement with this result, different researchers [41, 44, 45] reported that prevalence of brucellosis in sheep at animal and flock level varied significantly depending on production systems. The current finding was also in agreement with the observation of Lone et al. [46] who reported higher seroprevalence of brucellosis in unorganized extensive sector (14.14%) compared with the prevalence in intensive organized sheep farming (3.23%). This lower individual level seroprevalence in the breeding ranches can be correlated with good and controlled management practices and screening of male animals for brucellosis before using them for breeding purpose which could reduce the transmission of ovine brucellosis. Culling breeding females with reduced reproductive performances and with history of abortion could also reduce the risk of within-flock transmission/spread of brucellosis in intensively managed breeding ranches [47].

The higher prevalence in extensive small holder production may be due to the fact that, in this production system, there is free animal movement and aggregation of animals within common pastures and watering points which may increase the transmission of brucellosis from animal to animal or from contaminated environment [48–50].

Other possible risk factors for brucellosis related to extensive smallholder production in Ethiopia include ram sharing for breeding, which may result in venereal transmission of ovine brucellosis, and the herd composition in smallholder production, where sheep are mixed with cattle and goats, while in the breeding ranches only pure sheep flocks are kept for breeding purpose. This is in agreement with the findings by Holt et al. [51] and Megersa et al. [52] who reported that the risk of transmission of brucellosis significantly increased in mixed herds than in pure herds.

With regard to flock level seroprevalence, the present study revealed that all the eight sheep flocks examined in the breeding ranches were found to be 100% seropositive, while 19.92% of the flocks in the extensive production system were found to be seropositive. Similarly, Bayemi et al. [53] and Karimuribo et al. [54] reported higher seroprevalence of brucellosis in intensively managed flocks compared to flocks under extensive production. This could be related to large flock size; the number of individuals in each studied breeding ranch was more than 50 sheep per flock. Large flocks are more likely to have at least one positive sheep compared with small-sized flocks, which increased the transmission of brucellosis; in addition, in large flocks, there will be mass management practices that allow for closer contact between animals and make it more difficult to control transmission of brucellosis in large flocks.

Significantly higher seroprevalence was found in the midland both in individual level and flock level with prevalence of 9.07% (95% CI: 7.54-11.7,* P* < 0.01) and 4.5% (95% CI: 2.71-5.03,* P* < 0.05), respectively. The difference in seropositivity between the two agroclimates was found to be significantly variable (*P* < 0.01). This higher seroprevalence in the midland compared to the highland can be correlated with the increasing animal's concentration and contact due to shortage of grazing lands in midlands, which favor the transmission of brucellosis [55]. Additionally, the favorability of the midland agroclimates for survival and multiplication of* Brucella* organisms has been associated with the spread of brucellosis [55].

In this study, seroprevalence of ovine brucellosis was significantly higher (*P* < 0.001) in female (8.21%) than in male (3.01%) sheep under extensive production system. In agreement with current result, similar studies in Ethiopia [35, 56] and Bangladesh [57] reported higher seroprevalence in female sheep [57]. The higher prevalence in female than in male sheep under extensive farming may be due to the fact that female sheep in the study areas are usually reared for breeding and kept for a longer period within the flocks without culling, even though females had reduced reproductive performances and were at old age. Thus females have ample time for exposure to* Brucella *organisms and being source of infection for others [58]. The presence of erythritol in allantoic fluid during pregnancy favors the growth and propagation of* Brucella *organisms, thereby enhancing the susceptibility of female sheep to brucellosis [42, 56].

This study revealed that an increase in age was associated with increased seropositivity for ovine brucellosis in both production systems. Seroprevalences of brucellosis in sheep with age groups of 1-3 years and above 3 years were found to be almost the same: 5.9% and 6%, respectively. However, the prevalence was found to be significantly higher (*P* < 0.05) in sheep with 1-3 years (5.9%) compared to animals with 6 months-1 year age group (2.56%). In agreement with this, Cadmus et al. [59] reported no significant difference in seroprevalence in cattle that are above 3 years and 1–3 years old. A similar observation by Wesinwe et al. [34] has been reported in northeast Ethiopia. Contrary to this result, Matope et al. [60] reported that seroprevalence of brucellosis has been decreased with increasing age. The observed increasing seroprevalence rate with age in this finding may be probably due to the fact that sex hormones and erythritol that stimulate multiplication of* Brucella* organisms tend to increase in concentration with age and sexual maturity. Seroprevalence may also increase with age as a result of prolonged duration of antibody responses in infected animals and prolonged exposure to pathogen [61].

In breeding ranches, none of the sheep below 1 year of age were found to be seropositive but seroprevalence increased without significant difference from 0% to 0.08% in age group of 1-3 years. Significantly the highest seroprevalence (3%) was observed in above 3 years age group. In agreement with our findings, a study in sheep ranch by Aregawi et al. [62] reported seroprevalence of 0%, 0.38%, and 2.25% in sheep with age groups below 2 years, 2-3 years, and above 3 years, respectively.

In this finding, significantly higher proportion of seroprevalence was found in the flock category having more than 20 sheep per flock (37%, 95% CI: 31-44.53) than in those flocks with 11-20 sheep (27.8%, 95% CI: 21.8-30.3) and 1-10 sheep per flock (10%, 95% CI: 6.71-14.1), respectively. This study was in agreement with previous studies, which reported that brucellosis was associated with large herd size [63–65]. Similar result in Uganda showed that the majorities of seropositives were detected only in large and medium-sized flock [66]. Stocking density allows greater contact between animals and their environment, which increases the potential for exposure to infectious excretions and also increases the risk of exposure to* Brucella* infection especially following abortion [65]. Another explanation might be due to the fact that grazing in communal pastures may facilitate the contact between infected and uninfected flocks [64, 67].

In the current study, 40% (95% CI: 32.1-52.4) of seropositive pregnant ewes under smallholder extensive production were found with history of abortion, while 15.6% of seropositive ewes did not have a history of abortion and carried the pregnancy to full term. Seroprevalence was found to be significantly higher (*P* < 0.01) in ewes with history of abortion compared to pregnant ewes with no history of abortion. This result is consistent with a similar study in Nigeria by Boukary et al. [68] who reported seroprevalence of brucellosis associated with the incidence of abortions. Mahajan and Kulshreshtha [69] similarly reported significantly higher seroprevalence of ovine brucellosis in ewes with history of abortion (74.6%) than in ewes without abortion (20.37%). This result is generally in agreement with several authors who reported that the prevalence of brucellosis within flocks/herds is positively correlated with the incidence of abortions in females and abortion is the most obvious manifestation of brucellosis [70, 71]. However, only 6.25% (95% CI: 4.1-8.35) of seropositive ewes were with history of abortion in breeding ranches compared with the 40% seroprevalence of brucellosis in ewes with history of abortion under smallholder extensive farming. This result is in agreement with previous reports by different researchers [72–74]; free grazing and abortion have been identified as risk factors for brucellosis related to extensive system.

In the current study, a significant difference was observed in seropositivity between ewes that had previous history of retained fetal membrane, compared with those without retained fetal membrane in both production systems. However, seroprevalence of brucellosis was comparatively much higher in ewes with history of retained fetal membrane which are under smallholder production (50%,* P* < 0.01) than in ewes kept in breeding ranches (8.3%,* P* < 0.01). In agreement with this result, Chukwu [75] showed that brucellosis is frequently associated with retained fetal membrane. The higher seroprevalence of brucellosis in ewes under extensive smallholder production may be due to the fact that farmers in the study areas tend to keep ewes, even if they have history of abortion and retained fetal membrane because of the disease is subclinical in most animals. For these reasons, farmers seldom cull infected animals from their flocks, contributing to the high prevalence observed in this study.

## 5. Conclusions

The result of the present seroepidemiological survey shows that brucellosis is an important sheep disease and well-entrenched infection across the selected zones and districts of the eastern Amhara region with higher seroprevalence in the extensive mixed farming system. Infected sheep were found in all flocks of the three studied government breeding and multiplication ranches; therefore more efforts should be directed towards improving the animal health, biosecurity program, and regular screening in those ranches that are used for cross-breeding. In smallholder farms, 50% of the ewes with history of abortion were seropositive for brucellosis; as a result, the smallholder extensive management practices significantly support the spread of brucellosis in the study areas, especially in the midlands of the studied zones. These show that brucellosis is becoming an impediment to the exploitation of the huge small ruminant population of the traditional smallholder farmers of the Amhara region, hence the need for improved management systems and implementing appropriate control measures in smallholder production. Generally those control measures in intensively managed governmental ranches could account for the lower animal level seroprevalence reported in this study. Raising public awareness on zoonotic transmission of brucellosis associated with milk consumption and contact with aborted materials is recommended in the study areas.

## Figures and Tables

**Table 1:** Comparative analysis between Rose Bengal Plate Test (RBPT) and Complement Fixation Test (CFT) for the diagnosis of ovine brucellosis.

CFT result	RBPT result		Total	Prevalence (%)	Test agreement
	Positive (*n* = 120)	Negative (*n* = 2289)			
Positive (*n* = 118)	118	0	118	4.89	*k=* 0.958
Negative (*n* = 2291)	2	2289	2291	95.1	
Total	120	2289	2409		
Prevalence (%)	4.98	95			

**Table 2:** Individual level seroprevalence of ovine brucellosis in small holder farms and breeding ranches.

Production system	Individuals tested	RBPT (%)	CFT			FP (%)
			Positive (%)	*X* ^2^ (P)	95% CI	
Small holders	1838	110 (6)	108 (5.87)		3.83-7.31	2 (1.81)
Breeding ranches	571	10 (1.75)	10 (1.75)		1.57-3.05	-

Total	2409	120 (4.98)	118 (4.89)	35.07 (0.00)	3.24-6.9	2 (1.67)

**Table 3:** Flock level seroprevalence of ovine brucellosis in small holder farms and breeding ranches.

Production system	Flocks tested	RBPT (%)	CFT			FP (%)
			Positive (%)	*X* ^2^ (P)	95% CI	
Small holders	266	53 (19.9)	53 (19.9)		16.4-25.81	-
Breeding ranches	8	8 (100)	8 (100)		-	-

Total	274	61 (22.3)	61 (22.3)	41.95 (0.00)	18.03-29.17	-

**Table 4:** Individual level and flock level seroprevalence of brucellosis in the small holder sheep in relation to study areas and agroecology.

Zones	Districts	Individuals tested	Positive sera (%)	*X* ^2^ (P)	95% CI	Flocks tested	Positive sera (%)	*X* ^*2*^ (P)	95% CI
North Shewa		446	11 (2.3)		1.02-3.51	139	11 (7.91)		5.21-10.0
	Lalo Mider	225	6 (2.67)			73	7 (9.58)		
	Angolela	221	5 (2.26)			65	5 (7.69)		
South Wollo		901	55 (6.1)		4.83-7.41	75	25 (33.3)		28.53-40.1
	Legambo	515	22 (4.27)			36	11 (30.5)		
	Were Ilu	386	33 (8.54)			38	14 (36.8)		
North Wollo		459	42 (9.55)		7.91-12.4	52	17 (32.7)		27.21-37.0
	Delenta	294	23 (7.8)			27	10 (37.03)		
	Guba Lafto	165	19 (11.51)			25	7 (28)		
Total		1838	108 (5.87)	47 (0.00)	4.63-7.31	266	53 (19.9)	65.7 (0.00)	16.4-25.8
Agroecology									
Highland		1287	58 (4.5)		2.71-5.03	201	34 (16.9)		12.0-19.14
Midland		551	50 (9.07)		7.54-11.7	65	19 (29)		25.01-34.1
Total		1838	108 (5.87)	14.7 (0.001)	3.46-7.05	266	53 (19.9)	25.8 (0.012)	15.81-24.6

**Table 5:** Seroprevalence of brucellosis in sheep breeding and multiplication ranches kept under intensive production system.

Zone	Breeding ranches	Individuals tested	Positive (%)	95% CI	Flocks tested	Number of positives
North Shewa	Debre Berhan	257	6 (2.33)	2.01-2.57	3	3
	Amedguya	286	3 (1.0)	0.13-1.38	4	4
	Sheno ARC	28	1 (3.57)	2.84-5.18	1	1
Total		571	10 (1.75)	1.57-3.05	8	8 (100%)

**Table 6:** Seroprevalence of brucellosis in flock size and sex of the animals in relation to production system.

	Small holders farms				Breeding ranches			
	Number of tested	Positive (%)	*X* ^2^ (P)	95% CI	Number of tested	Positive (%)	*X* ^2^ (P)	95% CI
Sex								
Male	828	25 (3.01)		2.41-4.34	105	1 (0.95)		0.42-3.83
Female	1010	83 (8.21)		5.2-11.8	466	9 (1.93)		1.01-4.63
Total	1838	108 (5.87)	24.1 (0.00)	4.64-7.55	571	10 (1.75)	11.45 (0.16)	0.86-4.03
Flock size								
1-10	149	15 (10)		6.71-14.1	-	-	-	
11-20	61	17 (27.86)		21.8-30.3	-	-	-	
>20	56	21 (37)		31-44.53	8	8 (100)		-
Total	266	53 (19.92)	27.45 (0.000)	15.4-24.3	8	8 (100)	18.23 (0.045)	-

**Table 7:** Seroprevalence of brucellosis in relation to history of reproductive problem in breeding ranches and small holder production.

History of reproductive problems	Small holders farms				Breeding ranches			
	Number of tested	Positive (%)	*X* ^2^ (P)	95% CI	Number of tested	Positive (%)	*X* ^2^ (P)	95% CI
Abortion								
Present	55	22 (40)		32.1-52.4	16	1 (6.25)		4.1-8.35
Absent	218	34 (15.6)		12.4-18.71	337	6 (1.5)		0.79-3.5
Total	273	56 (20.5)	14.27 (0.001)	16.7-25.2	353	7 (1.7)	12.45 (0.000)	1.02-3.8
Retained fetal membrane								
Present	32	16 (50)		36.12-64.7	12	1 (8.3)		5.03-10.7
Absent	241	42 (17)		13.4-25.3	341	6 (1.7)		0.69-3.8
Total	273	56 (20.5)	26.21 (0.001)	18.3-29.08	353	7 (1.9)	8.030 (0.008)	0.8-4.02

## Data Availability

The data used to support the findings of this study are available from the corresponding author upon request.
